# Simultaneous Determination of Four Aldehydes in Gas Phase of Mainstream Smoke by Headspace Gas Chromatography-Mass Spectrometry

**DOI:** 10.1155/2019/2105839

**Published:** 2019-02-03

**Authors:** Xiaotao Zhang, Ruoning Wang, Li Zhang, Jianke Wei, Yibin Ruan, Weiwei Wang, Houwei Ji, Jian Liu

**Affiliations:** ^1^Technology Center, China Tobacco Guizhou Industrial Co., Ltd., Guiyang 550009, China; ^2^Minimal Invasive Center, Tumor Hospital, Sun Yat-Sen University, Guangzhou 510060, China

## Abstract

A method for simultaneous determination of acetaldehyde, propionaldehyde, acrolein, and crotonaldehyde in gas phase of cigarette mainstream smoke by headspace gas chromatography-mass spectrometry was developed and validated. Gas phase components of mainstream cigarette smoke were extracted with methanol, and then the samples were separated on a DB 624 (60 m, 0.32 mm x 1.8 mm) column, analyzed with headspace gas chromatography-mass spectrometry, and quantified by isotope internal standard. The linearities of acetaldehyde, propionaldehyde, acrolein, and crotonaldehyde were good (*R*^2^>0.992). The recoveries of acetaldehyde, propionaldehyde, acrolein, and crotonaldehyde were between 78.5% and 115%. The relative standard deviations were less than 10%. The limits of detection and limits of quantitation were 0.014 *μ*g/cigarette ~0.12 *μ*g/cigarette and 0.045 *μ*g/cigarette ~0.38 *μ*g/cigarette, respectively. The method had advantage of high sensitivity, it did not require derivatization of 2,4-dinitrophenylhydrazine and avoided a large number of adverse reactions during the process of derivation to improve the accuracy of result, and it was suitable for quantitative analysis of four aldehydes in gas phase of cigarette mainstream smoke.

## 1. Introduction

Smoking is a risk factor for lung diseases [1] and many cancers [2]. Cigarette mainstream smoke is a dynamic aerosol that contains more than 5,000 chemical constituents, containing particulate phase and a remaining gas phase [3]. The gas phase can pass into the bloodstream through the pulmonary circulation, which leads to acute and latent systemic actions [4]. Various carbonyl compounds present in the gas phase of cigarette smoke play a significant role in cigarette smoke toxicology [5], e.g., acetaldehyde, propionaldehyde, acrolein, crotonaldehyde, etc. These aldehydes react with nucleophilic targets in cells such as lipids, proteins, and DNA to form adducts. Those adducts may disturb cellular functions as well as damage proteins, nucleic acids, and lipids [6]. Acetaldehyde has been classified as a group 2B carcinogen for humans by the International Agency for Research on Cancer [7], while acrolein and crotonaldehyde have been classified as a group 3 carcinogen. It is important to quantitatively analyze the content of carbonyl compounds in the gas phase of cigarette mainstream smoke so as to understand smoke-related exposure estimates and systemic toxicity related to cigarette smoking.

For analyzing carbonyls, 2,4-dinitrophenylhydrazine (DNPH)-treated Cambridge filter pad (CFP) has been used to trap carbonyls in mainstream smoke generated from a smoking machine, and the levels of carbonyls are quantitatively determined by high-performance liquid chromatography (HPLC) [8, 11–15], HPLC coupled with mass spectrometry (LC–MS) [16], ultra-high-pressure liquid chromatography tandem mass spectrometry (LC–MS/MS) [9], or gas chromatography–mass spectrometry (GC–MS) [10, 17]. However, the process of DNPH-treated CFP was very labor-intensive, time-consuming, and tedious. The typical derivatization of carbonyls (e.g., DNPH) results in the formation of E and Z stereoisomers, which have different UV absorbance maxima. If a HPLC-UV method only measures a single isomer, or if the wavelength is not optimized for both isomers, carbonyl concentrations can be underestimated [18]. Recently, Zhao et al. [19] developed a method to rapidly detect acrolein and crotonaldehyde by a water-assisted atmospheric pressure chemical ionization tandem mass spectrometry (APCI-MS/MS). The method did not need any sample handling, but a modified APCI source to introduce sample directly into the ionization region and a 2 L glass container designed to make the standards gas by gasification of standards solution were needed. Sampson et al. [10] developed an automated volatile organic compounds determination method for mainstream smoke using solid phase microextraction (SPME) coupled with GC–MS. They used a gas sampling bag to collect and maintain vapor-phase smoke, and then it was homogenized with isotopically labeled analogue internal standards and sampled using gas-phase SPME. The method was less labor intensive and reduced sample handling.

Here, an impinger containing 20 mL methanol and cooled to a temperature of less than -70°C in a dry-ice/isopropanol bath was used to trapping acetaldehyde, propionaldehyde, acrolein, and crotonaldehyde in gas phase of mainstream smoke, then the impinger solution was analyzed by headspace gas chromatography–mass spectrometry (HS-GC-MS). It did not require derivatization of DNPH and avoided a large number of adverse reactions during the process of derivation to improve the accuracy of result.

## 2. Materials and Methods

### 2.1. Chemicals and Reagents

Acetaldehyde (1000 *μ*g/mL dissolved in methanol), acrolein (100 *μ*g/mL dissolved in methanol), and crotonaldehyde (1000 *μ*g/mL dissolved in acetonitrile) were purchased from AccuStandard corporation (New Haven, USA). Propionaldehyde (Purity, 98%) was purchased from TCI Shanghai Chemical Industry Co., LTD (Shanghai, China). Benzene-D6 (Purity, 98%) was purchased from J&K Scientific LTD (Beijing, China). Methanol (HPLC-grade) was purchased from TEDIA (Fairfield, USA). Water was purified by a milli-Q water purification system (Billerica, USA). Test cigarettes were purchased from China National Tobacco Corporation (Beijing, China).

### 2.2. Standard Solutions

Standard stock solutions: in a 10 mL volumetric flask, 100.0 milligrams of propionaldehyde was diluted to the mark with methanol. It was a solution containing 10.0 mg/mL propionaldehyde. Working solution was prepared with methanol by serial dilutions of stock solution to a concentration of 100 *μ*g/mL. Working solutions were prepared daily and stored at -20°C.

Internal standard (IS) stock solutions: in a 10 mL volumetric flask, 400.0 milligrams of benzene-D6 was diluted to the mark with methanol. It was a solution containing 40.0 mg/mL benzene-D6. Working solution was prepared with methanol by serial dilutions of stock solution to a concentration of 1 mg/mL.

### 2.3. Sample Preparation

Cigarettes were smoked on a smoking machine that had been fitted with impingers under ISO 3308 smoking regimes (35 mL; 2 s, 60 s). The mainstream cigarette smoke was passed through a CFP; the gas phase was cryogenically trapped in an impinger containing 20 mL methanol and cooled to a temperature of less than -70°C in a dry-ice/isopropanol bath. Then an aliquot of the smoke extract was syringe-filtered, and 100 *μ*L of samples was transferred to 20 mL vial and subjected to analysis by using HS-GC-MS.

### 2.4. HS-GC-MS Analysis

All the extractions were performed in 20 mL vials containing 0.1 mL of sample with a stirring speed of 250 rpm. The HS procedure was performed by using a Combi-PAL autosampler (Varian Pal Autosampler, Switzerland) and the Cycle Composer software (CTC Analytics System Software, Switzerland). The incubation time, extraction time, and temperature were 0.45 min, 10 min, and 90°C, respectively. Injection time was 0.5 min. A sample ring was used to inject, and the temperature of the sample ring is 160°C.

The TRACE 1310 GC (ThermoFisher, Waltham, USA) injector was held at 180°C. The oven temperature was held at 35°C (2 min) and then increased at 2°C/min to 80°C, and then increased at 20°C/min to 200°C (maintained 6 min). All mass spectra were obtained with a ThermoFisher ISQ LT instrument (Waltham, USA). The ion source was operated in the electron ionization (EI; 70 eV) mode. An Elite-624 capillary column (60 m × 0.32 mm I.D. × 0.18 *μ*m film thickness) (PerkinElmer, Waltham, USA) was used for the separation of the aldehydes. The samples were injected in the split mode, and the split ratio was 10. The flow rate of helium as carrier gas was 1.0 mL/min. The ion detector was set as follows: the transfer line and manifold temperatures were 220°C and 230°C, respectively. The qualitative ions, quantitative ions, and retention times of acetaldehyde, propionaldehyde, acrolein, crotonaldehyde, and benzene-D6 were shown in Table 1.

## 3. Results and Discussion

### 3.1. The Trapping Effect of Aldehydes

In order to collect the aldehydes in the gas phase of mainstream smoke, the trapping effect of aldehydes was investigated by using two tandem impingers containing 20 mL methanol and cooled to a temperature of less than -70°C in a dry-ice/isopropanol bath, and the results were shown in Table 2. As can be seen from Table 2, the content of acetaldehyde, propionaldehyde, acrolein, and crotonaldehyde in the second impinger was less than 1% that in the first impinger. The content of acetaldehyde, propionaldehyde, acrolein, and crotonaldehyde in the gas phase of mainstream smoke was almost completely captured by the first impinger. Therefore, an impinger containing 20 mL methanol and cooled to a temperature of less than -70°C in a dry-ice/isopropanol bath was used to trap the aldehydes in gas phase of mainstream smoke for following analysis.

### 3.2. Optimization of Extraction Time and Temperature

**Figure 1:**
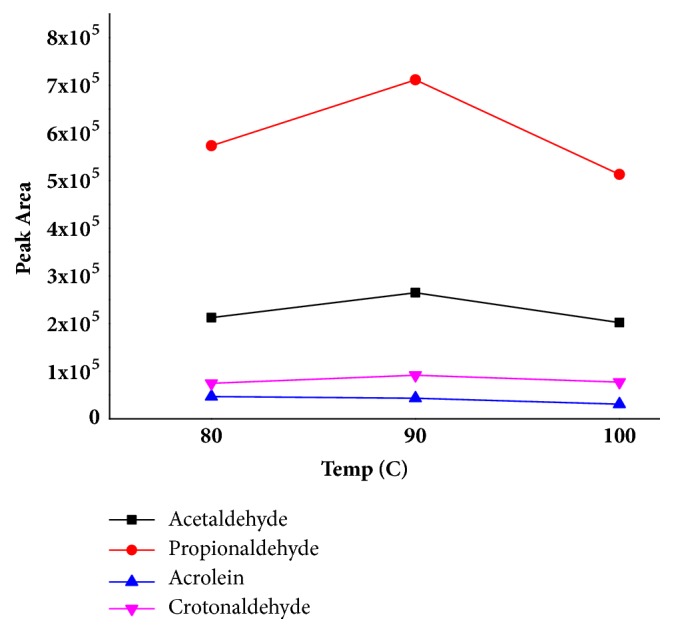
The influence of the HS extraction temperature on the peak area of each compound.

The influence of the HS extraction time (10 min, 15 min, and 20 min) on the peak area of each compound was investigated. The difference of peak areas of acetaldehyde, propionaldehyde, acrolein, and crotonaldehyde at different extraction time was not significant. So 10 min extraction time was selected for further analysis. In addition, the influence of the HS extraction temperature (80°C, 90°C, and 100°C) on the peak area of each compound was assessed. The result was shown in Figure 1. The peak areas of acetaldehyde, propionaldehyde, acrolein, and crotonaldehyde at 90°C were higher than that in the 80°C or 100°C. 90°C extraction temperature was used for further analysis.

### 3.3. Method Validation

**Figure 2:**
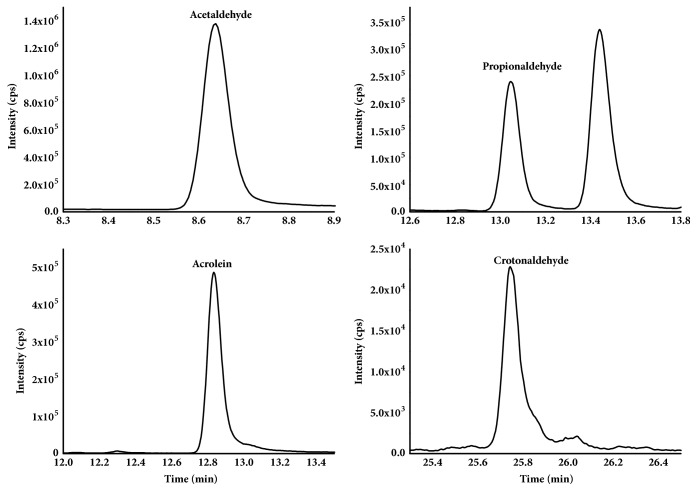
Extraction ion chromatograms of acetaldehyde, propionaldehyde, acrolein, and crotonaldehyde.

The linearity of the calibration curves was generated by performing linear regression of the ratio of peak area of analyte to the peak area of corresponding internal standards (y) onto concentration of analyte (x, ng/mL). Calibration standards in solvent at six concentrations were used for analysis of the linear relationship. The linear equations for acetaldehyde, propionaldehyde, acrolein, and crotonaldehyde were good with correlation coefficients (*R*^2^) ranging from 0.9921 to 0.9994 (Table 3). The limit of detection (LOD) and limit of quantitation (LOQ) were estimated with the signal/noise method using the integrated function of the Xcalibur software. LOD and LOQ were defined as the lowest analyte concentration that yielded a signal-to-noise ratio of 3 and 10 in real sample, respectively. The LODs for the acetaldehyde, propionaldehyde, acrolein, and crotonaldehyde were from 0.014 to 0.12 *μ*g/cig, and LOQs were from 0.045 to 0.38 *μ*g/cig. The extraction ion chromatography of acetaldehyde, propionaldehyde, acrolein, and crotonaldehyde was shown in Figure 2. And the concentrations of acetaldehyde, propionaldehyde, acrolein, and crotonaldehyde in Figure 2 were 62.6 *μ*g/cig, 12.0 *μ*g/cig, 15.7 *μ*g/cig, and 10.4 *μ*g/cig, respectively.

For analysis of recovery and precision, low, medium, and high concentrations of acetaldehyde, propionaldehyde, acrolein, and crotonaldehyde were spiked into the trapping solutions of 1R5F reference cigarettes (at least six replicates). The recoveries of targeted analytes in the real samples were determined by comparing the calculated amounts of targeted analytes from the spiked samples to the total spiked amounts of target analytes. The recoveries of acetaldehyde, propionaldehyde, acrolein, and crotonaldehyde ranged from 78.5% to 115% at three different spiked levels (Table 4). The precision was defined as relative standard deviation (*RSD*). The* RSDs* of all the analyses were less than 10%, which indicated that all the results were acceptable.

### 3.4. Sample Stability

**Figure 3:**
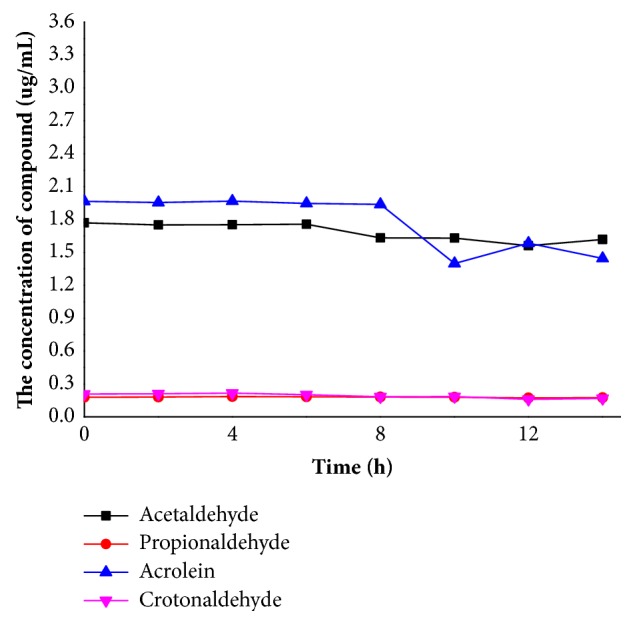
The stability of acetaldehyde, propionaldehyde, acrolein, and crotonaldehyde in methanol at room temperature.

Due to the high volatility and high reactive activity of acetaldehyde and acrolein, the stability of acetaldehyde, propionaldehyde, acrolein, and crotonaldehyde in methanol was investigated. The samples were placed at room temperature (20°C) and tested every 2 hours, and the results were shown in Figure 3. The contents of acetaldehyde, propionaldehyde, and crotonaldehyde changed little in 12 hours, but the content of acrolein was significantly reduced over 8 hours. This may be due to the high reaction activity of acrolein, so all the samples were analyzed within 8 hours.

### 3.5. Method Comparison

The aldehydes deliveries of the 3R4F cigarettes smoked under ISO smoking regimen were compared to further evaluate method performance. The results were shown in Table 5. Compared with the results published by Coresta method [8], the results had good consistency exception of acrolein. The reason for this may be that acrolein was a highly reactive unsaturated aldehyde. The results of this method were lower than that reported by Ding et al. [9] and Sampson et al. [10] with exception of crotonaldehyde.

### 3.6. Carbonyl Levels in Gas Phase of Mainstream Smoke from Domestic Cigarettes

**Figure 4:**
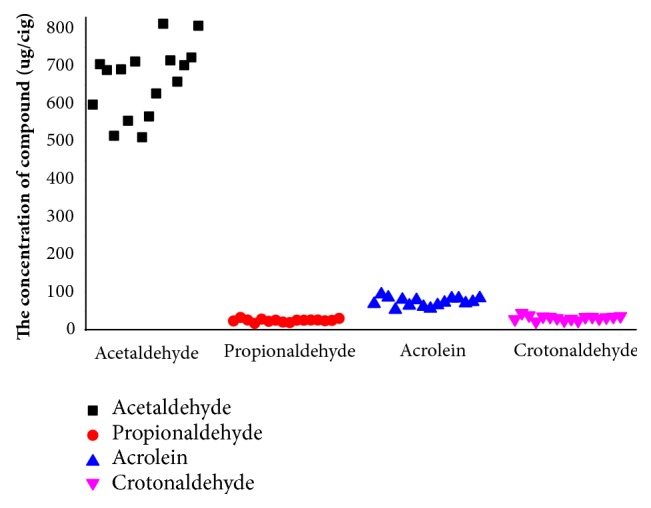
The contents of acetaldehyde, propionaldehyde, acrolein, and crotonaldehyde in gas phase of mainstream smoke.

The contents of acetaldehyde, propionaldehyde, acrolein, and crotonaldehyde in gas phase of mainstream smoke from 16 different brands of Chinese flue-cured tobacco samples were analyzed by using the proposed method. The results were shown in Figure 4. The average contents of acetaldehyde, propionaldehyde, acrolein, and crotonaldehyde in the gas phase of mainstream smoke from 16 different brands of Chinese flue-cured tobacco samples sold in China were 653.8, 25.66, 59.4, and 29.4 *μ*g/cig, respectively. However, the contents of acetaldehyde, propionaldehyde, acrolein, and crotonaldehyde in mainstream smoke of cigarettes sold in the United States were 1544.7, 169.2, 127.5, and 48 *μ*g/cig, respectively [9]. The concentrations of carbonyls in cigarette smoke sold in the United States were higher than that in cigarette smoke sold in China. The variation differences in the carbonyls deliveries of cigarette mainstream smoke could likely result from differences in product characteristics or tobacco types.

## 4. Conclusion

A method for simultaneous determination of acetaldehyde, propionaldehyde, acrolein, and crotonaldehyde in gas phase of cigarette mainstream smoke by HS-GC-MS was developed and validated. The method had advantage of high sensitivity, it did not require derivatization of DNPH and avoided a large number of adverse reactions (e.g., the formation of E and Z stereoisomers) during the process of derivation to improve the accuracy of result, and it was suitable for quantitative analysis of four aldehydes in gas phase of cigarette mainstream smoke.

## Figures and Tables

**Table 1:** Quantitation ions, confirmation ions, and retention times of the target compounds and their isotope internal standards.

Compound	Quantification ion (*m/z*)	Confirmation ion (*m/z*)	Retention time (min)
Acetaldehyde	44	43	8.64
Propionaldehyde	58	29	13.04
Acrolein	56	55	12.82
Crotonaldehyde	70	41	25.72
Benzene-D6	84	82	23.96

**Table 2:** The trapping effect of two impingers.

No. of impingers	Acetaldehyde (*μ*g/mL)	Propionaldehyde (*μ*g/mL)	Acrolein (*μ*g/mL)	Crotonaldehyde (*μ*g/mL)
1st	52.46	4.24	9.01	1.34
2nd	0.26	0.01	0.01	0.02

**Table 3:** Linear equations, LODs, and LOQs of acetaldehyde, propionaldehyde, acrolein, and crotonaldehyde (*n*=6).

Compound	Linear range (*μ*g/mL)	Linear equations	*R* ^2^	LOD (*μ*g/cig)	LOQ (*μ*g /cig)
Acetaldehyde	0.5~80	Y=0.00743342 x+0.000517293	0.9978	0.115	0.38
Propionaldehyde	0.055~14.08	Y=0.0171652 x+0.00075096	0.9994	0.014	0.045
Acrolein	0.49~58.8	Y=0.00847022x-0.0039118	0.9921	0.12	0.41
Crotonaldehyde	0.1~6.4	Y=0.00307451x-0.000137982	0.9952	0.03	0.10

**Table 4:** The recoveries and precisions (*RSDs*) of acetaldehyde, propionaldehyde, acrolein, and crotonaldehyde (*n*=6).

Compound	Sample (*μ*g/cig)	Spiked (*μ*g/cig)	Recovery (%)	Precision (%)
Acetaldehyde		80	105	3.45
	247	200	88.0	4.45
		400	78.5	1.72
Propionaldehyde		17.6	95.5	3.73
	11.4	35.2	89.6	1.07
		70.4	97.6	1.36
Acrolein		3.92	115	5.57
	16.9	15.7	102	2.15
		29.4	100.3	8.97
Crotonaldehyde		4	102	4.86
	10.8	16	84.0	7.93
		32	104	7.01

**Table 5:** Comparison of aldehydes in cigarette smoke with literature values (*μ*g/cig).

Compound	This method	Coresta Method [8]	Ding YS, et al. [9]	Sampson MM, et al. [10]
Acetaldehyde	524.6	552.0	659	620
Propionaldehyde	42.4	42.0	62.0	-
Acrolein	41.8	48.0	58.0	-
Crotonaldehyde	13.4	11.0	14.0	9.51

-: not reported in the literature.

## Data Availability

The data used to support the findings of this study are included within the article.
